# Differential Regulation of Morphology and Estrogen Receptor-Alpha Expression in the Vagina of Ovariectomized Adult Virgin Rats by Estrogen Replacement: A Histological Study

**DOI:** 10.1155/2016/1093512

**Published:** 2016-08-24

**Authors:** Ting Li, Yuanyuan Ma, Hong Zhang, Ping Yan, Lili Huo, Yongyan Hu, Xi Chen, Ting Li, Miao Zhang, Zhaohui Liu

**Affiliations:** ^1^Department of Obstetrics and Gynecology, Peking University First Hospital, 8 Xishiku Street, Beijing 100034, China; ^2^Animal Center Laboratory, Peking University First Hospital, 8 Xishiku Street, Beijing 100034, China; ^3^Department of Pathology, Peking University First Hospital, 8 Xishiku Street, Beijing 100034, China; ^4^Department of Nuclear Medicine, Peking University First Hospital, 8 Xishiku Street, Beijing 100034, China

## Abstract

*Background.* To determine the exact role of estrogen in vaginal tissue morphology and estrogen receptor-alpha (ER*α*) distribution in the vagina, which remains controversial.* Methods.* Sixty rats were randomly categorized: sham-operated (sham), ovariectomy (OVX), and four estradiol treatments (estradiol valerate at 0.4, 0.8, 1.6, and 3.2 mg/kg/day) for 2 weeks. Thereafter, vaginal samples were biopsied from the distal- and proximal-half portions. The percentage of ER*α*-immunoreactive cells and the ER*α* score were quantified using immunohistochemistry to assess changes in ER*α* expression and distribution.* Results.* OVX induced significant vaginal atrophy and organic index. Estrogen-replacement therapy (ERT) reversed vaginal atrophy. The vaginal distal-half areas showed lower ER*α*% than the proximal-half areas. The ER*α*% increased sharply 4 weeks after OVX, especially in the epithelial layer (*P* = 0.023). ERT elicited different degrees of reductions in tissues after the 2-week treatment, but the ER*α*% in only the epithelium recovered in parallel with that in the sham group (*P* = 0.001). The OVX group showed higher ER*α* histological scores than the sham group, and the distal-half area changed more evidently than the proximal-half area. ER*α* expression was nearly unchanged after ERT (*P* > 0.05).* Conclusions.* ERT is effective for treating obesity and vulvovaginal atrophy caused by hypoestrogenism and advancing age in menopausal women but cannot recover the distribution and expression of ER*α*.

## 1. Introduction

Estrogen plays central and pleiotropic roles in regulating behavior and function of the female reproductive processes. Hypoestrogenism or menopause has been implicated in female sexual dysfunction [1], which is associated with a variety of vulvovaginal atrophy symptoms, including vaginal dryness, dyspareunia, and irritation [2].

Menopause-related hormone therapy or other hormone therapy has been commonly used to treat menopause-related symptoms to prevent osteoporosis and cardiovascular disease and vulvovaginal atrophy symptoms [3]. The 2012 hormone-therapy position statement of The North American Menopause Society concluded that estrogen therapy (ET) is the most effective treatment for moderate or severe symptomatic vulvovaginal atrophy (e.g., vaginal dryness, itching, discomfort, and painful intercourse) [4]. However, the optimal regimen of ET required to achieve the desired relief is controversial, and some studies reported that low-dose systemic regimens may be inadequate for relieving vaginal symptoms [4, 5]. Estradiol valerate, a natural estrogen 17*β*-estradiol, is a synthetic 17-pentanoyl ester commonly used in clinical hormone treatment for menopausal symptoms, such as vaginal symptoms, hot flashes, and night sweats [6, 7].

The action of estrogen is primarily mediated via binding to intracellular estrogen receptors (ERs), which leads to transcriptional activation of estrogen-responsive genes and subsequent modification of cellular responses [8]. The mediation of estrogen action in the target organ is associated with not only the serum level, but also the subtypes and distribution of ERs [8, 9]. Therefore, the role of ET in treatment of vulvovaginal atrophy and improvement of sexual function through ERs in postmenopausal women needs further research.

A number of studies in rats as well as humans have shown that that ER*α* is widely distributed in the epithelium, muscularis, and stromal fibroblasts of the vagina, but the expression of ER*β* in the vagina is not yet well established [8, 10–13]. Some studies have reported low levels of ER*β* or its complete absence in the rat vagina [8, 14]. Moreover, estrogenic functions such as vaginal epithelial stratification and cornification are not observed in ER*α*-knockout mice following estrogen-replacement treatment (ERT), suggesting that ER*α* is the critical receptor for mediating specific vaginal responses commonly associated with ET [14, 15].

In the present study, we aimed to investigate the estrogen-ER interactions in the vagina by determining the distribution and expression of ER*α* following administration of different concentrations of estradiol valerate, by using immunohistochemistry. In addition, we examined the effects of ET on body weight and entire length as well as wet weight of the vagina, which have not been adequately addressed by previous studies in this field.

## 2. Materials and Methods

### 2.1. Ethical Approval

Guidelines of the National Institutes of Health for laboratory animal care were followed, and all experimental procedures and protocols were approved by the Ethics Committee of Peking University First Hospital (Beijing, China).

### 2.2. Animals

Sixty female virgin Sprague-Dawley rats aged 2-3 months (225 ± 15 g) were purchased from Beijing Vital River Laboratory Animal Technology Co., Ltd. (Beijing, China). Rats were raised in clusters of 4-5 per cage in a climate- and humidity-controlled environment (25 ± 1°C; relative humidity, 50%) with a 12L : 12D artificial cycle (lights on at 1000 hours), receiving food and water ad libitum in the Animal Center Laboratory of Peking University First Hospital.

### 2.3. Experimental Design

Rats were randomly assigned to 3 groups: the sham-operated group (sham, *n* = 10), the ovariectomy group (OVX, *n* = 10), and the ovariectomy + estradiol valerate group (*n* = 40; Bujiale; 1 mg/tablet; Guandong branch of Bayer Healthcare, Guangdong, China). Within a week of arrival, all rats were anesthetized by i.p. injection of 1% pentobarbital sodium (40 mg/kg). Rats in the OVX and ovariectomy + estradiol valerate groups underwent bilaterally ovariectomized using the ventral approach [16], while sham rats underwent bilateral laparotomy without ovariectomy. The serum estradiol levels were measured using an immunoradiometric assay on postoperative day 28 to ensure successful OVX and verify that the plasma estrogen levels were below the physiological range (baseline E2 in intact rats: 17 ± 2 to 21 ± 2 pg/mL [17]).

Four weeks after surgery, the sham and OVX groups received once-daily administration of 10 mL/kg saline by lavage for 2 weeks. At this point, the ovariectomy + estradiol valerate group was further divided into 4 groups and administered the following doses of estradiol valerate everyday between 08:30 hours and 09:30 hours for 2 weeks: 0.4 mg/kg (E1), 0.8 mg/kg (E2), 1.6 mg/kg (E3), and 3.2 mg/kg (E4) (*n* = 10, resp.). Thus, in total, there were 6 groups of rats. A previous study using identical doses of medication showed that once-daily administration of 0.8 mg/kg estradiol valerate dosage by lavage for 2 weeks to OVX rats resulted in restoration of normal physiological estrogen levels [16]. In this study, all drug doses were adjusted based on the weight of rats prior to administration.

Fourteen days after treatment, the rats were anesthetized, as described above. Whole-vagina samples were biopsied from all groups, as described by Ting et al. [18]. Animals were killed by asphyxiation.

### 2.4. Body Weight Gain and Vaginal Index Measurement

Vagina samples were rapidly biopsied and weighed to determine the vaginal index. The body weights and entire vaginal length were measured in a standardized manner by one investigator. The body weight gains were calculated and recorded, and the vaginal indexes were calculated according the following formula:

(1) $$\begin{array}{c} \text{Vaginal index}=\frac{\text{Vaginal wet weight}\left(\text{g}\right)}{\text{Body weight}\left(\text{g}\right)}\times 100\% \end{array}$$

(see [19]).

### 2.5. Morphological and Histopathological Changes

All biopsies were immersed immediately in 10% neutral buffered formalin, cut transversely into two halves of equal length, and embedded in paraffin using conventional histopathological methods. Five-micron cryosections were obtained perpendicular to the longitudinal axis of the vagina from the center of the two halves [20]. Each section was stained with hematoxylin and eosin (H&E) for routine microscopy. Morphological and histopathological changes of the vaginal wall were assessed.

### 2.6. ER*α* Immunohistochemical Analyses

Slides were incubated with 3% peroxide-methanol at room temperature for 20 min to block endogenous peroxidase, and with 1% diluted goat serum to block nonspecific binding sites. ER*α* (Dako, Glostrup, Denmark) was added following the avidin-biotin-peroxidase procedure using an EnVision Automated Immunostainer (Dako). Slides were then counter-stained with Gill's hematoxylin (Santa Cruz, CA, USA). Negative control sections were treated as described above, but the primary antibody was replaced by 0.01 M phosphate-buffered saline.

### 2.7. ER*α* Distribution Assessment

Randomly selected sections of each immunostained set from each half of the vaginal tissue were blind-coded and evaluated independently by two experienced pathologists using a digital microscope (BH2; Olympus, Tokyo, Japan). The index of the percentage of ER*α*-immunoreactive (ER-ir) cells (ER*α*%) in individual layers (epithelium, lamina propria, and muscularis) of the vaginal wall was calculated, and 100 cells were counted in each labeled area.

### 2.8. Semiquantification of ER*α* Score

Constantine's Criteria [21] for histochemistry scores were applied as follows: (a) for ER*α*% staining: 0, 0–10%; 1, 11–40%; 2, 41–70%; and 3, 71–100%. (b) For intensity of immunostaining: 0, no staining; 1, light yellow; 2, yellow-brown; and 3, dark brown. The ER*α* histological score is the sum of the two scores above (a + b): 0-1, negative (−); 2, weakly positive (+); 3-4, positive (++); 5-6, strongly positive (+++). The total ER*α* histological score of all layers in the vagina was calculated as the sum of the 3 histological scores of the epithelium, lamina propria, and muscularis; the maximum score was 18 points.

### 2.9. Statistical Analysis

Statistical analyses were conducted using SPSS 13.0 (SPSS, Chicago, IL, USA). All data for continuous variables are presented as the means ± standard deviation (SD). Parametric data were compared by Student's* t*-test or one-way analysis of variance, and nonparametric data were compared by Mann-Whitney* U* test or Kruskal-Wallis test. The level of significance was set at *P* < 0.05 in all analyses.

## 3. Results

### 3.1. Effects of Estrogen on Body Weight, Vaginal Wet Weight, Vagina Length, Vaginal Index, and the Morphology of the Vaginal Wall

The body weight gain, vaginal wet weight, vagina length, and vaginal index of all groups are shown in Table 1. The body weight gain in the OVX group was significantly higher than that in the sham group (*P* = 0.004). Body weight gain reduced to different degrees after supplementation of estrogen for 2 weeks (*F* = 9.124, *P* < 0.001; Table 1). The wet weight of the vagina in the OVX group decreased significantly (60%) as compared to the sham group and returned to the normal weight after supplementation of estrogen for 2 weeks, irrespective of the dose of estradiol valerate (*F* = 9.124, *P* < 0.001; Table 1). The vaginal index showed similar changes as vaginal wet weight (*F* = 9.643, *P* < 0.001; Table 1). These results indicate that ET may slow down the weight gain of OVX rats and relieve vaginal atrophy.

The vaginal length was the longest in the OVX group (*P* = 0.048) among all groups. Although the differences were statistically significant, the changes in the length were very small, ranging from 0.05 mm to 2 mm, and may be regarded as no clinical significance.

**Figure 1:**
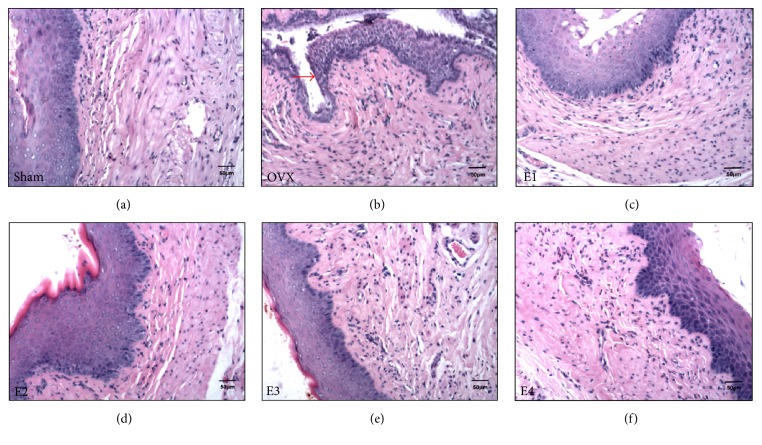
H&E immunostaining of vaginal tissue sections. (a)–(f) show H&E immunostaining of vaginal tissue sections from sham, OVX, E1, E2, E3, and E4 groups, respectively. The atrophic vaginal squamous epithelium in the OVX group is indicated by a red arrow in (b), exhibiting reduced epithelial stratification and cornification. Scale bar represents 50 *μ*m (200x magnification). H&E, hematoxylin & eosin.

H&E staining revealed that the epithelial layer in the sham group comprised thick stratified squamous epithelium with obviously keratinized differentiation. Rich supply of capillaries was observed throughout the lamina propria and thick muscularis. OVX was found to induce significant vaginal atrophy, showing thinner epithelial and muscular layers, less epithelial proliferation, and keratinized differentiation. The nuclei of nearly all epithelial cells (essentially basal cells) were changed into short column nuclei and showed loss of cytoplasm. The thickness of the muscularis appeared to decrease, along with an increase in the number of neutrophils in the lamina propria. Thus, ERT reversed the vaginal atrophic processes (Figures 1(a)–1(f)).

### 3.2. OVX Results in Increased Vaginal Distribution of ER*α*

**Figure 2:**
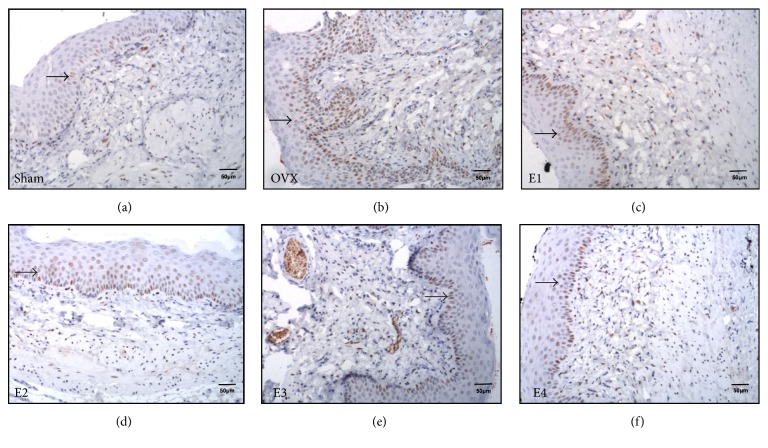
ER*α* immunostaining of vaginal tissue sections. (a)–(f) show ER*α* immunostaining of vaginal tissue sections from sham, OVX, E1, E2, E3, and E4 groups, respectively. ER*α*-immunoreactive cells are indicated by small black arrows. The staining is primarily nuclear in the vaginal stratified squamous epithelium (basal, para-basal, and intermediate cell layers) and in the stromal fibroblasts or nonvascular smooth muscle cells in the lamina propria. Scale bar represents 50 *μ*m (200x magnification). ER*α*, estrogen receptor-*α*.

In the intact sham rats, ER*α* staining in the vaginal stratified squamous epithelium was primarily nuclear and was localized in the basal (strongest), para-basal, and intermediate cell layers (Figures 2(a)–2(f)), but the superficial cells were not stained, similar to that reported in women of reproductive age [12]. Examination of the lamina propria and muscularis revealed that the majority of ER*α*-ir cells were stromal fibroblasts or nonvascular smooth muscle cells. Qualitative analysis of the vaginal wall in the sham group revealed that the ER*α*% values throughout the epithelium, lamina propria, and muscularis were lower in the distal-half areas than in the proximal-half areas (*P* = 0.256, *P* = 0.035, and *P* = 0.042, resp.; Table 2).

We examined the ER*α* distribution in all layers of the vaginal wall before and 6 weeks after OVX (Table 3). An increase in the ER*α*% values was observed for all the layers following OVX but reached statistical significance only in the epithelium (*P* = 0.023). Notably, the proportion of ER*α*-ir cells increased sharply by almost 5-fold in the distal-half areas, while the increase in the ER*α*% was less pronounced in the proximal-half areas (2–5 fold).

### 3.3. Effect of Estrogen on Vaginal Distribution of ER*α*%

Following 2 weeks of sustained estradiol valerate administration after OVX, compared to that in OVX and sham group, ER*α*% fluctuated within the range of low dose (E1) to extra-high dose (E4) of estradiol valerate for treated animals in various layers of the vaginal wall in both the distal- and proximal-half areas (Table 3). Among the 3 layers of both the distal- and proximal-half areas, the ER*α*% in only the epithelial layer recovered with different concentrations of estrogen in parallel with that in the sham group.

### 3.4. Effect of Estrogen on the Semiquantitative Histological Score of ER*α*

**Figure 3:**
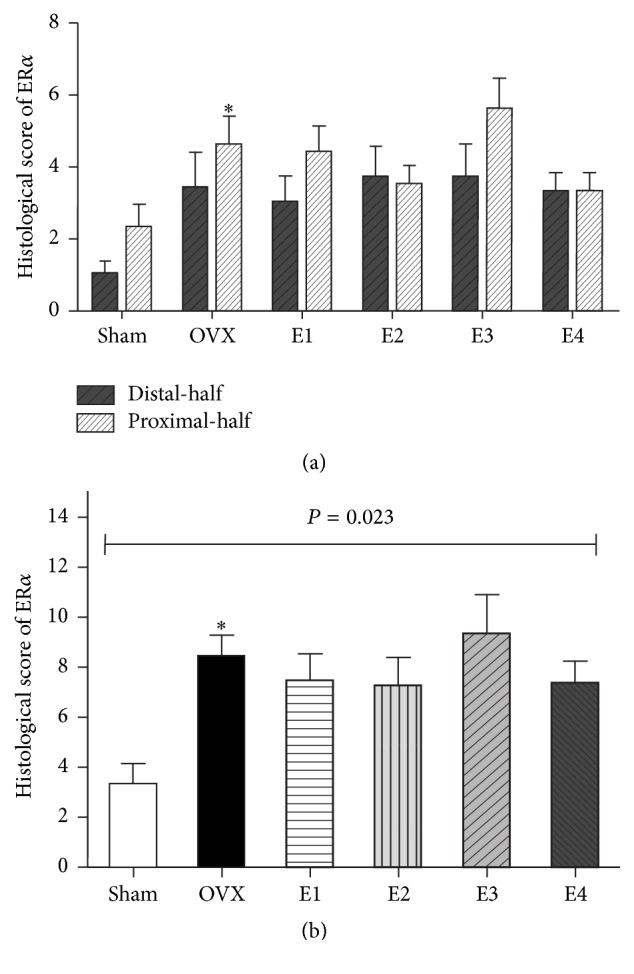
Histological scores of ER*α* in the distal- and proximal-half areas. (a) presents the histological scores of ER*α* in the distal- and proximal-half areas in each group. (b) presents the histological scores of ER*α* in the whole vagina in each group. ^*∗*^ *P* = 0.039: ER*α* score in the whole vagina is significantly higher in the OVX group than in the sham group (Mann-Whitney* U* test).

A comparison of the histological score of ER*α* using Constantine's Criteria [21] was a composite score that is based on histologic assessment of ER*α*-ir cells and the staining intensity (Table 4). Staining intensity was generally higher in stromal cells of the vaginal lamina propria than in the epithelial cells, suggesting that the former may be more receptive to estrogen action [20]. The ER*α* scores for OVX animals in the distal- or proximal-half areas or whole vagina increased to different degrees as compared to the sham group, and statistical significance was observed in the whole vagina (*P* = 0.023). The ER*α* scores showed a more evident increase for the distal-half sections than for the proximal-half sections (Figure 3(a)).

After 2 weeks of continuous ERT, the ER*α* scores showed similar changes as ER*α*%, fluctuating within the range of low dose (E1) and extra-high dose (E4) of estradiol valerate for treated animals in both the distal- and proximal-half areas, as compared to the OVX and sham groups. There was no statistically significant difference in the ER*α* score for the whole vagina between each treatment group and the OVX group (all *P* > 0.05; Figure 3(b)).

## 4. Discussion

In this study, we established bilateral-ovariectomized rat models and then administered different doses of estradiol valerate, ranging from subphysical to supraphysiological levels, to mimic the improvement in obesity and vaginal structure from the vulvovaginal atrophy, due to ET in postmenopausal women. The vaginal index is a good parameter for measurement of genital atrophy in experimental animals [19]. The reproductive organs of OVX rats showed degenerative changes, and the vaginal index in OVX rats decreased remarkably due to the lack of the protective beneficial effects of estrogen. In agreement with the results from the morphological observations, our histopathological examination showed that estrogen deficiency after menopause predisposed rats to the onset of vulvovaginal atrophy, reduction in the thickness of the epithelial and muscular layers, failure of keratinized differentiation, and decrease in the number of capillaries in the lamina propria, as was reported in previous studies [15, 22–24]. Moreover, the sudden increase in body weight in OVX rats may be related to estrogen regulation of serum leptin, adiponectin, insulin resistance, lipid metabolism, and other factors [25–27]. All estradiol doses, including the subphysical dose (E1), induced hyperplastic epithelium with thickened vaginal mucosa and presence of vacuolated cells, greatly improving the karyoplasmic ratio of the epithelial cells, which is consistent with the results of a previous study [24]. Epithelial ER*α* can integrate estradiol via growth factor signaling to regulate squamous differentiation and cell proliferation [22]. The vaginal morphology, vaginal index, vaginal histopathologic changes, and body weight of rats recovered to normal after ET, suggesting that a subphysical dose of estradiol valerate is a good treatment option for reversing postmenopausal obesity and dyslipidemia, thereby alleviating menopause-induced vaginal atrophy.

Estrogen actions are mainly mediated by classic ERs [28, 29], and the two major forms of ERs identified thus far are ER*α* [30] and ER*β* [31]. Previous studies have reported loss of vaginal epithelial stratification and cornification in ER*α*-knock-out mice even after estradiol treatment, suggesting that ER*α* is a critical receptor for regulating specific vaginal responses to estrogen [15, 16].

Data on the comparative distribution of ER*α* in the distal- and proximal-half parts of vagina are limited. In the present study, using immunohistochemistry, we systematically investigated the ratio of ER*α*-ir cells and staining intensity in the vagina of rats under different doses of systematic estrogen, ranging from subphysical to supraphysiological levels. ER*α* showed differences in its regional distribution throughout the distal- and proximal-half areas of the vaginal wall in rats. Although the number of ER*α*-ir cells in the proximal-half areas was more than that in the distal-half areas, the expression density shown by the histological scores in the proximal-half areas was less than that in the distal-half areas. These receptor divergences can be attributed to the embryological origin, urogenital sinus, and Müllerian ducts of the distal- and proximal-half areas.

OVX triggers compensatory ER*α* upregulation in all layers of both the distal- and proximal-half areas of the vaginal wall. Interestingly, OVX resulted in a uniform compensatory increase (>twofold) in ER*α* expression, while ER*α* expression was nearly unchanged (nonsignificant) with all doses of estradiol. These findings are consistent with those of several previous studies [12, 22, 32–34]. These results could be due to the following reasons: (a) ER*α* is mainly distributed within the vaginal epithelium; due to estrogen deficiency-induced epithelial atrophy, the number of the ER*α*-negative superficial cells of the stratified epithelium may have decreased so drastically that the percentages of the residual ER*α*-positive basal cells, para-basal cells, and intermediate cells in epithelium increased significantly. (b) A negative-feedback mechanism between estrogen and ERs may explain the compensatory overexpression of ER*α* [35, 36]. (c) Destruction of the cellular structure caused by estrogen deficiency leads to the exposure of intracellular ER*α*. Estradiol valerate therapy can only repair damaged mucous membrane but is incapable of retrieving exposed ER*α* inside the cells. We postulate that ER*α* exposure caused by estrogen deficiency or menopause cannot be completely reversed by ET, as serine 118 and serine 167 located in the AF-1 domain of ER*α* are persistently phosphorylated by estrogen expression, and this phosphorylation can lead to cancerous lesions in the vagina or other genital organs later in life [32]. Thus, the optimal dose of estrogen for treatment of vaginal dryness and dyspareunia symptoms in perimenopausal women needs further exploration.

Several of our findings are contrary to those reported in other studies [33, 34]. The reason for the discrepancy is that there is a “time window” for systematic ET and decrease in estrogen levels. When establishing the OVX model, we found that even 14 days after OVX, serum estradiol levels in model rats did not reach 17 ± 2 to 21 ± 2 pg/mL, which is the baseline E2 range in intact rats [37], until 28 days after OVX the rats were just successfully modeling. Most studies that began treatment as early as 2–14 days after OVX confirmed these findings by vaginal smear, which examines the functional changes, not the biological changes. The declining serum estradiol level had not reached a “trigger point” to induce compensatory upregulation of ER*α*; therefore, the most “idle” ER*α* would gradually degrade, following the low estradiol expression. Furthermore, since the expression of ER*α* is closely related to the level of estrogen, we could not choose only one or two administered doses of estrogen to reveal the estrogen-ER interaction.

## 5. Limitations

Some limitations should be considered. Whether the phenomenon and effect of estrogen observed in “menopausal” rat model in the current study is in accordance with that in women during perimenopausal period needs to be investigated. Research regarding the mechanism of estrogen in the vagina is in its initial stages, and further studies should focus on other subsequent signaling pathways and mechanisms of ET in vulvovaginal atrophy symptoms. Future studies also should investigate the effect that other steroid hormone and receptors, such as androgens and androgen receptor, mainly believed to be involved in female sexual function.

## 6. Conclusions

In summary, the present study provides morphologic and biochemical data in support of the role of estrogen in the vagina. Systemic estrogen administration at subphysiological levels clearly ameliorated obesity and vulvovaginal atrophy in rats but could not normalize compensatory ER*α* upregulation, suggesting that this approach may be useful in treating a series of postmenopausal symptoms caused by vaginal atrophy. Premature or early menopause is the appropriate “critical period” or “time window” for estradiol treatment. Further research is needed to choose the optimal regimen and timing of systemic ET and to understand how it applies to individual women.

## Figures and Tables

**Table 1:** Effects of estrogen on vaginal morphologic parameters (*α* = 0.05, two-tailed).

Location	Sham	OVX	E1	E2	E3	E4	*F*	*P*
Body weight gain (g)	39.10 ± 5.01	89.40 ± 9.25	74.80 ± 13.49	89.00 ± 6.19	68.60 ± 4.47	42.80 ± 8.14	6.926	<0.001^b^
Length (cm)	2.19 ± 0.06	2.38 ± 0.04	2.22 ± 0.07	2.33 ± 0.04	2.26 ± 0.06	2.15 ± 0.07	2.411	0.048^a^
Wet weight (g)	0.25 ± 0.14	0.15 ± 0.01	0.25 ± 0.02	0.25 ± 0.01	0.24 ± 0.01	0.24 ± 0.01	9.124	<0.001^b^
Vaginal index (%)	0.092 ± 0.006	0.047 ± 0.003	0.082 ± 0.007	0.078 ± 0.003	0.079 ± 0.004	0.085 ± 0.005	9.643	<0.001^b^

^a^ *P* < 0.05 and  ^b^ *P* < 0.001 (one-way analysis of variance).

**Table 2:** Index of the percentage of vaginal ER*α*-immunoreactive cells according to location in sham rats (*α* = 0.05, two-tailed).

Location	Subjects	Distal-half	Proximal-half	*t*	*P*
Epithelium	10	6.70 ± 1.41	12.90 ± 5.60	1.214	0.256
Lamina propria	10	5.50 ± 2.17	16.70 ± 5.76	2.484	0.035^a^
Muscularis	10	4.70 ± 2.13	18.50 ± 5.38	2.371	0.042^a^

^a^ *P* < 0.05 (paired sample *t*-test).

**Table 3:** Index of the percentage of vaginal ER*α*-immunoreactive cells (ER*α*%) according to location in all groups (*α* = 0.05, two-tailed).

Location	Subjects	Sham	OVX	E1	E2	E3	E4	*F*	*P*
Proximal-half	10								
Epithelium		12.90 ± 5.60	45.00 ± 11.59	17.00 ± 5.18	13.20 ± 2.61	15.50 ± 3.29	10.80 ± 1.73	4.678	0.001^b^
Lamina propria		16.70 ± 5.76	33.70 ± 10.53	38.10 ± 7.37	27.50 ± 7.00	56.50 ± 8.89	32.00 ± 7.93	2.683	0.031^a^
Muscularis		18.50 ± 5.38	29.00 ± 7.22	30.50 ± 7.97	20.70 ± 5.82	54.50 ± 8.77	36.20 ± 7.67	3.223	0.013^a^

Distal-half	10								
Epithelium		6.70 ± 1.42	33.50 ± 10.49	18.50 ± 3.73	19.90 ± 6.64	18.30 ± 6.42	15.00 ± 1.83	2.116	0.077
Lamina propria		5.50 ± 2.18	25.00 ± 8.06	20.50 ± 8.06	37.50 ± 8.07	27.80 ± 7.89	12.50 ± 2.91	3.125	0.015^a^
Muscularis		4.70 ± 2.13	19.44 ± 9.07	10.70 ± 4.81	26.50 ± 7.71	33.50 ± 7.96	20.50 ± 4.50	2.698	0.030^a^

^a^ *P* < 0.05 and  ^b^ *P* < 0.001 (one-way analysis of variance).

**Table 4:** Histological scores of ER*α* according to location in all groups (*α* = 0.05, two-tailed).

Location	Sham	OVX	E1	E2	E3	E4	*Z*	*P*
Proximal-half	2.30 ± 0.62	4.60 ± 0.28	4.40 ± 0.70	3.50 ± 0.50	5.60 ± 0.83	4.00 ± 0.84	10.720	0.057
Distal-half	4.70 ± 2.13	19.44 ± 9.07	10.70 ± 4.81	26.50 ± 7.71	33.50 ± 7.96	20.50 ± 4.50	9.231	0.1
Whole	3.30 ± 0.82	8.00 ± 1.28	7.40 ± 1.08	7.20 ± 1.12	9.30 ± 1.55	7.30 ± 0.87	13.033	0.023^a^

^a^ *P* < 0.05 (Kruskal-Wallis test).
